# Effects of an amino acid mixture on alcohol metabolism and alcohol-related symptoms in healthy adults

**DOI:** 10.1038/s41598-026-35178-1

**Published:** 2026-01-08

**Authors:** Hyeongyeong Kim, Hyung Joo Suh, Kisoo Han, Nari Kim, Eun Young Jung, Joon Young Shim

**Affiliations:** 1 Department of Integrated Biomedical and Life Science, Graduate School, Korea University, Seoul, 02841 Republic of Korea; 2 Transdisciplinary Major in Learning Health Systems, Department of Healthcare Sciences, Graduate School, Korea University, Seoul, 02841 Republic of Korea; 3 Neo Cremar Co. Ltd, Seoul, 05702 Republic of Korea; 4 Department of Home Economic Education, Jeonju University, Jeonju, 55069 Republic of Korea

**Keywords:** Amino liver, Alcohol metabolism, Alcohol-related symptoms, Acetaldehyde, Clinical trial, Randomized controlled trial, Diseases, Gastroenterology, Health care, Medical research

## Abstract

Alcohol consumption is associated with metabolic burden and a range of alcohol-related symptoms. Amino acids have been suggested to support hepatic metabolism and recovery following alcohol consumption. This study investigated the effects of Amino Liver (AL), an amino acid mixture, on alcohol metabolism and alcohol-related symptoms in healthy adults. In a randomized, double-blind, placebo-controlled, crossover clinical trial, healthy adults consumed AL or placebo prior to alcohol consumption. Blood alcohol and acetaldehyde concentrations were measured at predefined time points up to 13 h after alcohol consumption. Alcohol-related symptoms were assessed using the Alcohol Hangover Severity Scale (AHSS). Safety parameters were also evaluated. AL intake was associated with a more rapid reduction in blood alcohol and acetaldehyde concentrations compared with placebo. Participants who received AL reported lower severity of alcohol-related symptoms, particularly fatigue and cognitive difficulty, at 13 h after alcohol consumption. No clinically significant adverse effects were observed. The findings suggest that AL supplementation is associated with favorable modulation of alcohol metabolism and reduced severity of alcohol-related symptoms. Although further studies are required to confirm these findings and clarify underlying mechanisms, AL may represent a potential nutritional approach for supporting alcohol metabolism following alcohol consumption. Trial registration: Clinical Research Information Service (CRIS), Republic of Korea (KCT0010612). Registered 10 June 2025. Retrospectively registered.

**Supplementary Information:**

The online version contains supplementary material available at 10.1038/s41598-026-35178-1.

## Introduction

Alcohol consumption is a widespread social and cultural practice around the world, yet it is often accompanied by undesirable physiological aftereffects, most notably hangover symptoms^1,2^. Hangovers are present as a constellation of physical and cognitive impairments—including fatigue, nausea, headache, dizziness, and reduced concentration—that emerge once the intoxicating effects of alcohol subside^3–5^. These symptoms are primarily attributed to the accumulation of acetaldehyde, a toxic intermediate of alcohol metabolism, as well as to downstream effects such as oxidative stress, neuroinflammation, and metabolic disruption^6,7^. Delayed clearance of acetaldehyde exacerbates systemic toxicity and intensifies hangover severity, making it a critical target for physiological intervention^8,9^.

Despite the global prevalence of hangovers, there remains no universally accepted or consistently effective treatment. A variety of remedies—including herbal extracts, antioxidants, vitamins, and hydration therapies—have been marketed or investigated as potential hangover interventions, yet most have produced inconclusive or anecdotal results^10–12^. Among the more promising candidates, amino acid supplementation—particularly with branched-chain amino acids (BCAAs)—has attracted attention for its role in hepatic detoxification, ammonia metabolism, and mitochondrial energy production^13–16^. However, most prior studies have examined BCAAs in isolation, without investigating their potential synergistic effects when combined with other functionally complementary amino acids. Thus, the broader therapeutic potential of multi-amino acid formulations remains underexplored despite compelling biochemical rationale.

Emerging biochemical and clinical evidence suggests that amino acids beyond BCAAs—specifically L-arginine, L-methionine, and L-alanine—may provide complementary metabolic benefits in the context of alcohol metabolism and hangover recovery. L-arginine enhances hepatic detoxification by supporting the urea cycle and promoting nitric oxide synthesis, which improves blood flow and toxin clearance^17,18^. L-methionine, a sulfur-containing amino acid, plays a pivotal role in glutathione synthesis and aldehyde detoxification, supporting the enzymatic activity of aldehyde dehydrogenase^19,20^. L-alanine, a gluconeogenic precursor, helps replenish energy stores depleted by alcohol metabolism, thereby addressing fatigue—one of the most reported hangover symptoms^21,22^. Together, these amino acids target distinct but interconnected pathways affected by alcohol, providing a compelling foundation for a multi-component therapeutic strategy.

To address the current lack of clinically validated hangover interventions, this study evaluated the safety and efficacy of a novel multi-component amino acid mixture, Amino Liver (AL), composed of BCAAs, L-arginine, L-methionine, and L-alanine. While previous studies have focused on BCAAs alone, few have assessed the potential synergistic effects of combining multiple amino acids with complementary metabolic roles. This study employed a randomized, double-blind, placebo-controlled, crossover clinical design to determine whether AL could enhance alcohol metabolism, promote acetaldehyde clearance, and reduce the subjective severity of hangover symptoms in healthy adults. We hypothesized that AL supplementation would enhance alcohol metabolism, lower blood levels of alcohol and acetaldehyde, and reduce the severity of hangover symptoms in healthy adults.

## Materials and methods

### Participants

Participants were recruited through local newspaper advertisements and online platforms between July 1 and December 31, 2024. Interested individuals underwent preliminary screening to determine eligibility based on the following criteria: aged between 19 and 49 years; not currently taking medications that could interfere with alcohol metabolism; no history of alcohol abuse or alcohol-related diseases; body mass index (BMI) between 18.5 and 25 kg/m²; and a consistent pattern of moderate alcohol consumption (e.g., 1–2 standard drinks per week). Exclusion criteria included pregnancy or lactation; a history of severe medical conditions, including cardiovascular, hepatic, renal, metabolic, neurological, or psychiatric disorders; or known disorders affecting alcohol metabolism. All participants provided written informed consent. After screening 33 candidates, 23 healthy adults met the inclusion criteria and were enrolled. Each participant attended two separate laboratory sessions, spaced at least 7 days apart to ensure adequate washout and prevent carry-over effects. However, 2 individuals withdrew before completing the second session due to personal reasons; thus, a total of 21 participants successfully completed both sessions.

### Procedures

This study employed a randomized, double-blind, placebo-controlled, crossover clinical design. This trial was retrospectively registered with the Clinical Research Information Service (CRIS), Republic of Korea (KCT0010612). The random allocation sequence was generated by computer using a simple randomization method, and the assignment order (AL or placebo first) was managed by a researcher not involved in data collection. The randomization list was implemented by a study coordinator using sequentially numbered, opaque containers to ensure allocation concealment. Accordingly, participants and investigators remained blinded to the intervention assignment throughout the study. To maintain double blinding, the investigational products were labeled with unique randomization codes. The allocation codes were sealed and securely managed by the principal investigator and were not disclosed until completion of the study, except in the event of a serious adverse event. No unblinding occurred during the study period.

The AL formulation and matching placebo were developed and manufactured by Neo Cremar Co. Ltd (Seoul, Republic of Korea). Both capsules were identical in appearance, weight, and packaging, and were supplied in coded containers prepared by the manufacturer. The AL formulation contained BCAAs (L-leucine, L-isoleucine, and L-valine), L-arginine, L-methionine, and L-alanine, selected for their roles in enhancing alcohol metabolism and liver function. The detailed composition and dosage of each ingredient in the Amino Liver formulation and the placebo are provided in Supplementary Table S1.

Participants arrived at the laboratory at 8:00 AM and consumed a standardized breakfast. Two hours later, they ingested three capsules (400 mg per capsule) of either AL or placebo, taken with 100 mL of water, 30 min prior to alcohol consumption. Participants then consumed an alcoholic beverage (25% alcohol by volume, equivalent to 0.78 g of alcohol per kg body weight) within 30 min, accompanied by a standardized snack (20 shrimp pieces, 60 kcal). Two hours after alcohol consumption, participants were permitted an additional 100 mL of water but were otherwise required to fast for the following 4 h.

Throughout the study, participants underwent physical examinations and laboratory assessments to monitor vital signs and detect any adverse events, which were tracked via structured interviews and questionnaires.

**Fig. 1:**
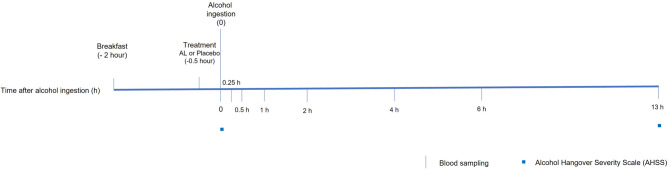
Study timeline and experimental procedure. Schematic overview of the randomized, double-blind, placebo-controlled crossover study design. Participants consumed Amino Liver (AL) or placebo 30 min prior to alcohol consumption. Alcohol was administered at a dose of 0.78 g/kg body weight. Blood samples were collected at baseline and at 0.25, 0.5, 1, 2, 4, 6, and 13 h after alcohol consumption to assess blood alcohol and acetaldehyde concentrations. Alcohol-related symptoms were evaluated using the Alcohol Hangover Severity Scale (AHSS) 13 h after alcohol consumption.

Figure 1 illustrates the study timeline on the experimental day, including the timing of treatment administration, alcohol consumption, blood sampling, and symptom assessment.

### Measurements

Vital signs—including systolic and diastolic blood pressure (SBP and DBP)—as well as liver function markers such as aspartate aminotransferase (AST), alanine aminotransferase (ALT), and gamma-glutamyl transpeptidase (γ-GTP) were measured at baseline to confirm participant health status. Thirteen hours after alcohol consumption, blood pressure and additional biochemical parameters were reassessed. All biochemical analyses were performed using a Dri-Chem 3500 Chemistry Analyzer (Fujifilm, Kanagawa, Japan).

Blood samples were collected at predefined time points (baseline, 0.25, 0.5, 1, 2, 4, 6, and 13 h) following alcohol consumption. To minimize volatilization, blood samples were processed promptly after collection, with immediate serum separation. Serum ethanol concentrations were measured using a commercial Ethanol Assay Kit (BM-ETH-100, BIOMAX, Guri, Republic of Korea), which is based on an alcohol dehydrogenase–mediated enzymatic reaction. Serum acetaldehyde concentrations were determined using an Acetaldehyde Assay Kit (ab112113, Abcam, Cambridge, UK) employing an enzymatic colorimetric method. All assays were conducted according to the manufacturers’ instructions.

Alcohol-related symptoms were assessed using the Alcohol Hangover Severity Scale (AHSS), which evaluates common symptoms such as fatigue, nausea, dizziness, concentration difficulty, and heart palpitations. Each symptom was rated on a scale from 0 (none) to 10 (extreme). The AHSS was administered before alcohol consumption and again 13 h after alcohol consumption.

### Statistical analysis

All randomized participants who completed both intervention phases were included in the final per-protocol analysis, and no missing data were reported. No subgroup or sensitivity analyses were prespecified or performed.

Statistical analyses were performed using SPSS version 21 (SPSS Inc., Chicago, IL, USA). Descriptive statistics were used to calculate means and standard errors of the mean (SEM). Paired t-tests were conducted to assess within-subject differences in blood alcohol concentration, acetaldehyde levels, and hangover symptom scores between the AL and placebo treatments. Additionally, repeated measures analysis of variance (ANOVA), followed by Bonferroni-adjusted pairwise comparisons, was used to evaluate temporal changes in outcomes. All tests were two-tailed, and statistical significance was defined as *p* < 0.05. As this study was exploratory in nature, no formal sample size calculation was performed; the final sample size was determined based on feasibility.

### Trial registration and ethics approval

The study was conducted in accordance with the Declaration of Helsinki, approved by the institutional review board of Jeonju University (approval number: jjIRB-240730-HR-2024-0609). Written informed consent was obtained from all participants prior to study enrollment. The trial was registered with the Clinical Research Information Service (CRIS), Republic of Korea (registration number: KCT0010612), first posted on 10/06/2025. The registration was performed retrospectively, after the enrollment of participants had commenced.

## Results

### Participants characteristics

The baseline characteristics of the 21 participants enrolled in the study are presented in Table 1. The cohort consisted of 10 males and 11 females, with a mean age of 32.71 ± 1.77 years. The average weekly alcohol consumption was 172.22 ± 40.83 g, and the mean age at first alcohol use was 19.62 ± 0.17 years. Participants reported engaging in physical activity approximately 1.90 ± 0.34 times per week, with each session lasting an average of 58.57 ± 9.58 min. Anthropometric data indicated a mean height of 171.57 ± 1.99 cm and a mean weight of 68.12 ± 2.54 kg, resulting in a mean BMI of 22.96 ± 0.44 kg/m², which falls within the healthy range. Mean systolic and diastolic blood pressures were 116.10 ± 2.01 mmHg and 70.95 ± 1.19 mmHg, respectively. Liver function parameters, including AST (23.90 ± 2.61 U/L), ALT (19.33 ± 1.38 U/L), and γ-GTP (30.52 ± 3.27 U/L), were all within normal reference ranges. These findings confirm that the participants were in good general health and exhibited no signs of hepatic dysfunction, making them suitable candidates for evaluating the effects of amino acid supplementation on alcohol metabolism and hangover severity. All participants received the assigned interventions as scheduled, and no protocol deviations or missed administrations occurred during the trial.

**Table 1:** Baseline demographic and physiological characteristics of participants.

Category	Parameters	Value
Demographics	Gender (Male/Female)	10/11
	Age (years)	32.71 ± 1.77
	Weekly alcohol consumption (g/week)	172.22 ± 40.83
	Age at first alcohol use (years)	19.62 ± 0.17
	Exercise frequency (times/week)	1.90 ± 0.34
	Duration per exercise session (min)	58.57 ± 9.58
Vital Signs	Height (cm)	171.57 ± 1.99
	Weight (kg)	68.12 ± 2.54
	BMI (kg/m²)	22.96 ± 0.44
	Systolic Blood Pressure (mmHg)	116.10 ± 2.01
	Diastolic Blood Pressure (mmHg)	70.95 ± 1.19
Liver Function	AST (U/L)	23.90 ± 2.61
	ALT (U/L)	19.33 ± 1.38
	γ-GTP (U/L)	30.52 ± 3.27

Healthy adults (*n* = 21) enrolled in a randomized crossover trial of Amino Liver supplementation. Data are expressed as mean ± standard error of the mean (SEM).

### Blood alcohol concentrations and acetaldehyde levels

**Fig. 2:**
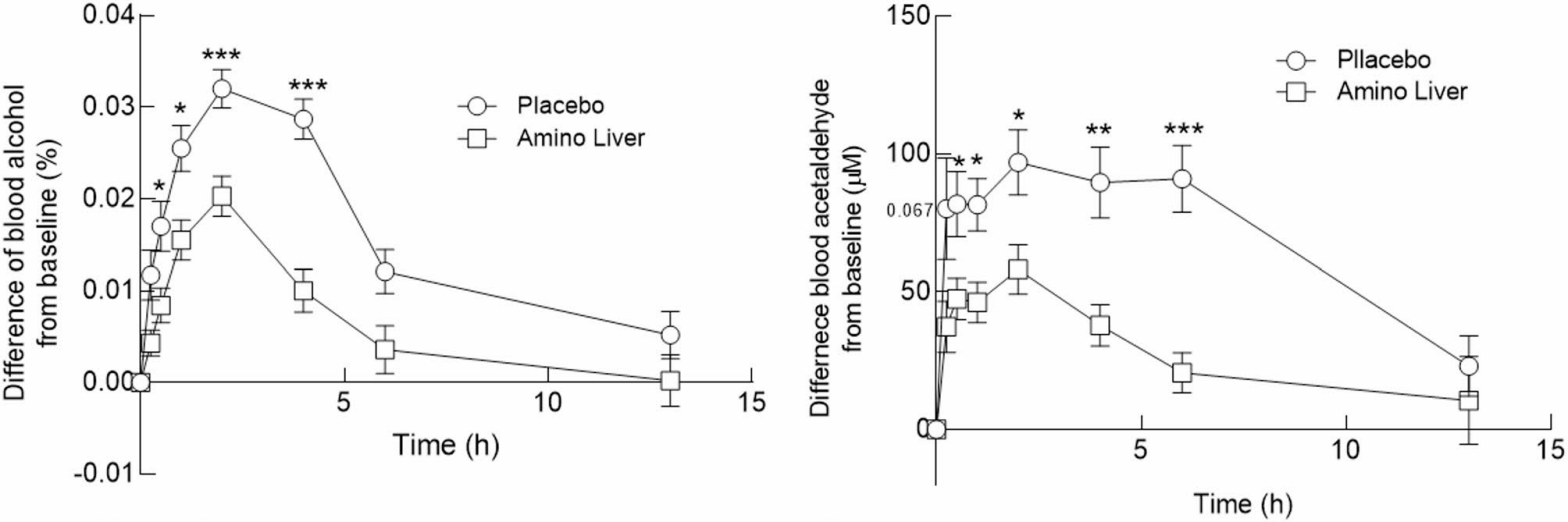
Blood alcohol and acetaldehyde concentrations after supplementation. Mean blood alcohol and acetaldehyde concentrations measured at baseline, 0.25, 0.5, 1, 2, 4, 6, and 13 h after alcohol consumption. Changes in blood alcohol concentrations and acetaldehyde levels following alcohol consumption in the Amino Liver (AL) and placebo treatments. Data are expressed as mean ± standard error of the mean (SEM). ^*^p < 0.05, ^**^p < 0.01, ^***^p < 0.001 compared to placebo.

Figure 2 illustrates the time-course changes in blood alcohol and acetaldehyde levels following alcohol consumption, comparing the AL and placebo treatments. Analysis revealed a significant main effect of treatment of blood alcohol concentration (*F* (1, 20) = 14.28, *p* < 0.001), as well as a significant treatment x time interaction (*F* (7, 140) = 5.12, *p* < 0.001). Post-hoc paired *t*-tests showed that blood alcohol concentrations were significantly lower in the AL treatment compared to the placebo treatment at 30 min (*t* (20) = 2.14, *p* = 0.0444), 1 h (*t* (20) = 2.43, *p* = 0.0243), 2 h (*t* (20) = 3.92, *p* = 0.0009), and 4 h (*t* (20) = 5.85, *p* < 0.001) after alcohol consumption.

For acetaldehyde levels, a significant main effect of treatment (*F* (1,20) = 11.56, *p* = 0.003) and a significant treatment x time interaction (*F* (7,140) = 3.85, *p* = 0.001) were observed. Paired comparisons demonstrated significantly lower acetaldehyde concentrations in the AL treatment at 30 min (*t* (20) = 2.42, *p* = 0.0249), 1 h (*t* (20) = 2.28, *p* = 0.0107), 2 h (*t* (20) = 2.42, *p* = 0.0247), 4 h (*t* (20) = 3.49, *p* = 0.0022), and 6 h (*t* (20) = 5.85, *p* = 0.00001). Collectively, these results indicate that AL supplementation was associated with faster reductions in blood alcohol and acetaldehyde concentrations over time compared with placebo.

### Hangover severity

Hangover severity was assessed using the AHSS, in which participants rated 12 common symptoms 13 h after alcohol consumption. Analysis revealed a significant main effect of treatment on the total AHSS score (*F* (1,20) = 4.35, *p* = 0.041). As shown in Table 2, the total AHSS score was significantly lower in the AL treatment (1.19 ± 0.36) compared to the placebo group (4.10 ± 1.27) (*t* (20) = 2.18, *p* = 0.041), indicating that AL effectively mitigated the overall severity of hangover symptoms.

**Table 2:** Hangover symptom severity (AHSS scores) after alcohol consumption.

Outcome	Treatment			ΔMean (AL-Placebo) (95% CI)	*P* value	
	Placebo (*n* = 21)		Amino Liver (AL) (*n* = 21)			
Total score	4.10 ± 1.27	1.19 ± 0.36		−2.91 (−4.12, −1.70)		0.041
Fatigue	1.43 ± 0.36	0.52 ± 0.18		−0.91 (−1.43, −0.39)		0.038
Apathy: lack of interest/concern	0.33 ± 0.19	0.00 ± 0.00		−0.33 (−0.41, −0.25)		0.102
Concentration problems	0.52 ± 0.21	0.05 ± 0.05		−0.47 (−0.56, −0.38)		0.045
Clumsiness	0.19 ± 0.11	0.05 ± 0.05		−0.14 (−0.19, −0.09)		0.246
Confusion	0.29 ± 0.12	0.05 ± 0.05		−0.24 (−0.30, −0.18)		0.077
Thirst	0.76 ± 0.34	0.33 ± 0.16		−0.43 (−0.63, −0.31)		0.273
Sweating	0.05 ± 0.05	0.05 ± 0.05		+ 0.00 (−0.03, 0.03)		1.000
Shivering	0.00 ± 0.00	0.00 ± 0.00		+ 0.00 (0.00, 0.00)		1.000
Stomach pain	0.29 ± 0.19	0.05 ± 0.05		−0.24 (−0.29, −0.19)		0.247
Nausea	0.14 ± 0.07	0.05 ± 0.05		−0.09 (−0.13, −0.05)		0.305
Dizziness	0.10 ± 0.09	0.05 ± 0.05		−0.05 (−0.09, −0.01)		0.657
Heart pounding	0.00 ± 0.00	0.00 ± 0.00		+ 0.00 (0.00, 0.00)		1.000

Symptoms were measured 13 h after alcohol consumption in healthy adults who participated in a randomized crossover trial receiving Amino Liver (AL) or placebo. Data are presented as mean ± standard error of mean (SEM) for each treatment (*n* = 21). Mean differences with 95% confidence intervals were calculated using paired t-tests. Abbreviations: AHSS, Alcohol Hangover Severity Scale.

Symptom-level analyses indicated that fatigue (*t* (20) = 2.21, *p* = 0.038) and concentration difficulties (*t* (20) = 2.14, *p* = 0.045) were significantly reduced under the AL treatment. Other symptoms, including nausea, headache, and dizziness, showed numerically lower scores following AL supplementation; however, these differences did not reach statistical significance. Overall, these findings indicate that AL supplementation was associated with reduced severity of alcohol-related symptoms.

### Safety and biochemical markers

To evaluate the safety of AL supplementation, a variety of physiological and biochemical markers were assessed before and 13 h after alcohol consumption. As shown in Table 3, no significant differences were observed between the AL and placebo treatments in systolic blood pressure (117.38 ± 1.84 mmHg vs. 116.10 ± 1.96 mmHg; *t* (20) = 0.13, *p* = 0.901) or diastolic blood pressure (71.33 ± 0.97 mmHg vs. 70.95 ± 1.16 mmHg; *t* (20) = 0.15, *p* = 0.882). Liver enzyme levels remained stable across both conditions. The AL treatment showed ALT of 21.90 ± 3.19 U/L, AST of 28.00 ± 6.48 U/L, and γ-GTP of 24.75 ± 4.15 U/L, with no significant differences compared to the placebo treatment (ALT: 22.75 ± 3.04 U/L, *t* (20) = 0.19, *p* = 0.852; AST: 23.65 ± 1.71 U/L, *t* (20) = 0.64, *p* = 0.530; γ-GTP: 24.45 ± 3.56 U/L, *t* (20) = 0.05, *p* = 0.958). In addition, no significant differences were observed in blood urea nitrogen (BUN: 15.30 ± 0.37 mg/dL vs. 16.26 ± 0.54 mg/dL, *t* (20) = 1.47, *p* = 0.158) or glucose levels (103.10 ± 3.25 mg/dL vs. 107.10 ± 3.39 mg/dL; *t* (20) = 0.84, *p* = 0.411). Collectively, these results indicate that AL supplementation was well tolerated and was not associated with adverse effects on hepatic, renal, cardiovascular, or metabolic parameters following alcohol consumption.

**Table 3:** Physiological and biochemical safety parameters after alcohol consumption.

Parameters	Treatment			ΔMean (AL-Placebo) (95% CI)	*P* value
	Placebo (*n* = 21)		Amino Liver (AL) (*n* = 21)		
Systolic Blood Pressure (mmHg)	116.10 ± 1.96	117.38 ± 1.84		+ 1.28(−4.15, 6.71)	0.901
Diastolic Blood Pressure (mmHg)	70.95 ± 1.16	71.33 ± 0.97		+ 0.38(−2.68, 3.44)	0.882
ALT (U/L)	22.75 ± 3.04	21.90 ± 3.19		−0.85(−9.76, 8.06)	0.852
AST (U/L)	23.65 ± 1.71	28.00 ± 6.48		+ 4.35(−9.19, 17.89)	0.530
γ-GTP (U/L)	24.45 ± 3.56	24.75 ± 4.15		+ 0.30(−11.75, 11.35)	0.958
BUN (mg/dL)	16.26 ± 0.54	15.30 ± 0.37		−0.96(−2.28, 0.36)	0.158
Glucose (mg/dL)	107.10 ± 3.39	103.10 ± 3.25		−4.00(−13.49, 5.49)	0.411

Parameters were measured 13 h after alcohol consumption in healthy adults who participated in a randomized crossover trial receiving Amino Liver (AL) or placebo. Data are expressed as mean ± standard error of the mean (SEM) for each treatment (*n* = 21). Mean differences with 95% confidence intervals were calculated using paired t-tests. Abbreviations: AST, aspartate aminotransferase; ALT, alanine aminotransferase; **γ-**GTP, gamma-**g**lutamyl transpeptidase; BUN, blood urea nitrogen.

## Discussion

This study aimed to evaluate the effects of AL supplementation on alcohol metabolism and alcohol-related symptoms. The findings suggest that AL intake was associated with a more rapid reduction in blood alcohol and acetaldehyde concentrations compared to placebo. In addition, participants who received AL reported lower severity of alcohol-related symptoms, particularly fatigue and cognitive difficulty. These observations support the potential benefits of combining BCAAs with functionally complementary amino acids such as L-arginine, L-methionine, and L-alanine. This formulation may provide a synergistic approach to supporting alcohol metabolism and post-intoxication recovery, consistent with previous reports on amino acid-based interventions for hepatic protection and metabolic regulation^13,16,19^.

Amino acid combinations enriched with BCAAs enhance mitochondrial efficiency and ATP production while protecting hepatic cells from ethanol-induced injury^16^. These findings are in line with earlier theoretical insights by Cynober^23^, who highlighted the role of amino acids in maintaining nitrogen balance, energy homeostasis, and cellular repair—particularly under conditions of physiological stress. Together, these studies reinforce the rationale for combining multiple functionally distinct amino acids in a single formulation such as AL, which delivers multi-targeted support for detoxification, antioxidant defense, and energy restoration. This comprehensive mode of action may account for the observed benefits of AL compared to BCAA supplementation alone.

Each of the additional amino acids, namely L-arginine, L-methionine, and L-alanine, contributes uniquely to enhancing alcohol metabolism. L-arginine facilitates hepatic detoxification via its role in the urea cycle and promotes nitric oxide production, which improves hepatic blood flow and supports toxin clearance^17,18^. L-methionine, a sulfur-containing amino acid, plays a key role in the synthesis of glutathione, a crucial antioxidant involved in neutralizing acetaldehyde and mitigating oxidative stress. Cha et al.^19^ demonstrated that methionine enhances the activity of alcohol-metabolizing enzymes, including ALDH, while Ali et al.^24^ emphasized its protective effect against alcohol-induced lipid peroxidation. Additionally, L-alanine plays a critical role in gluconeogenesis, helping to restore energy levels depleted during alcohol metabolism^21,25^. By combining these functionally diverse amino acids, AL provides a comprehensive system of metabolic support that addresses multiple pathways disrupted by alcohol consumption.

The synergistic effects of BCAAs and the additional amino acids in AL not only enhance alcohol metabolism but also offer a more holistic approach to reducing hangover severity. For instance, Zhang and Ren^26^ demonstrated L-arginine’s protective effects against alcohol-induced hepatotoxicity through its enhancement of detoxification pathways and reduction of oxidative stress. Similarly, L-methionine boosts ALDH activity and supports glutathione synthesis—both essential for acetaldehyde clearance^27,28^. These mechanisms are further supported by Kim et al.^14^, who reported that sulfur-containing amino acids such as methionine activate the nuclear factor erythroid 2-related factor 2 (Nrf2) antioxidant pathway, contributing to systemic protection against alcohol-related oxidative damage.

Several important limitations of this study should be considered when interpreting the findings. Although the Alcohol Hangover Severity Scale (AHSS) was used to assess symptoms commonly associated with hangover, the present study may not fully capture classic next-day hangover, which typically occurs when blood alcohol concentration approaches zero and involves multifactorial mechanisms such as dehydration, immune activation, and hormonal changes. Given the relatively low peak blood alcohol concentration observed in this study and the timing of symptom assessment, the symptoms evaluated here may primarily reflect early alcohol-related responses rather than classic hangover.

In addition, genetic polymorphisms affecting alcohol metabolism, particularly aldehyde dehydrogenase 2 (ALDH2), were not assessed. This limitation is especially relevant in East Asian populations, where reduced ALDH2 activity is prevalent and has been strongly associated with acetaldehyde accumulation and acute alcohol-related symptoms such as flushing^29^. Therefore, the observed effects of Amino Liver may, at least in part, reflect modulation of alcohol metabolism and associated symptoms in individuals with reduced acetaldehyde clearance.

Despite these limitations, the present findings suggest that the amino acid formulation was associated with lower systemic exposure to alcohol and acetaldehyde and with reduced severity of alcohol-related symptoms. Future studies incorporating genetic stratification and alternative symptom assessment time points will be important to further clarify the effects of Amino Liver on alcohol metabolism and post-intoxication symptoms. Additionally, the relatively small sample size and homogeneous participant characteristics may limit the generalizability of the findings.

Overall, the present study suggests that AL supplementation is associated with favorable modulation of alcohol metabolism and with reduced severity of alcohol-related symptoms. The combined actions of the included amino acids—supporting hepatic detoxification, antioxidant defense, and energy homeostasis—may contribute to these effects. Although further studies are required to confirm these findings and clarify underlying mechanisms, AL may represent a promising nutritional approach for supporting alcohol metabolism and alleviating alcohol-related discomfort.

## Supplementary Information

Below is the link to the electronic supplementary material.

Supplementary Material 1

## Data Availability

The datasets generated and/or analyzed during the current study are available from the corresponding author on reasonable request.

## Abbreviations

AHSS: Alcohol Hangover Severity Scale
AL: Amino Liver
ALDH: Aldehyde Dehydrogenase
ALT: Alanine Aminotransferase
AST: Aspartate Aminotransferase
BMI: Body Mass Index
BUN: Blood Urea Nitrogen
GTP: Gamma-Glutamyl Transpeptidase
SEM: Standard Error of the Mean
SPSS: Statistical Package for the Social Sciences
