# Supplementary material for: Effects of an amino acid mixture on alcohol metabolism and alcohol-related symptoms in healthy adults
Source: Sci Rep. 2026 Jan 8;16:4845. doi: 10.1038/s41598-026-35178-1 (PMC12873346; doi:10.1038/s41598-026-35178-1)
Supplement: Supplementary file 1 — Supplementary Material 1 [file 41598_2026_35178_MOESM1_ESM.docx]

**Supplementary Table S1.**

Composition and dosage of Amino Liver and placebo

| Component | Ingredient | Amount (mg) | Compositions (%) |
| --- | --- | --- | --- |
| Amino Liver | L-Leucine | 104.4 | 26.1 |
|  | L-Isoleucine | 52.0 | 13.0 |
|  | L-Valine | 52.0 | 13.0 |
|  | L-Arginine | 69.6 | 17.4 |
|  | L-Methionine | 41.6 | 10.4 |
|  | L-Alanine | 17.2 | 4.3 |
|  | Other component(s) * | 63.0 | 15.8 |
|  | **Total** | 400.0 | 100.0 |
| Placebo | Resistant starch | 400.0 | - |

* Other component(s) include inert excipients added to standardize capsule weight and maintain blinding consistency with the placebo, including resistant starch.
